# Epidemiology of childhood and adolescent cancer in Bangladesh, 2001–2014

**DOI:** 10.1186/s12885-016-2161-0

**Published:** 2016-02-15

**Authors:** Mohammad Sorowar Hossain, Mamtaz Begum, Md Mahmuduzzaman Mian, Shameema Ferdous, Shahinur Kabir, Humayun Kabir Sarker, Sabina Karim, Salma Choudhury, Asaduzzaman Khan, Zohora Jameela Khan, Henrike E. Karim-Kos

**Affiliations:** Faculty of Basic Sciences, Bangladesh University of Health Sciences, Darus Salam, Mirpur-1, Dhaka, 1216 Bangladesh; National Institute of Cancer Research and Hospital, Dhaka, Bangladesh; Centre for Excellence, University of Dhaka, Dhaka, Bangladesh; Talent Hub, Banani, Dhaka, Bangladesh; ASHIC Foundation, Dhaka, Bangladesh; Dhaka Medical College and Hospital, Dhaka, Bangladesh; School of Health and Rehabilitation Sciences, University of Queensland, Brisbane, Australia; Department of Public Health, Erasmus MC University Medical Centre, P.O. Box 2040, 3000 CA Rotterdam, The Netherlands

**Keywords:** Bangladesh, Cancer, Childhood, Adolescent, Leukaemia, ALL, Retinoblastoma, Incidence

## Abstract

**Background:**

Cancer burden among children and adolescents is largely unknown in Bangladesh. This study aims to provide a comprehensive overview on childhood and adolescent cancers and to contribute to the future strategies to deal with these diseases in Bangladesh.

**Methods:**

Data on malignant neoplasms in patients aged less than 20 years diagnosed between 2001 and 2014 (*N* = 3143) in Bangladesh was collected by the National Institute of Cancer Research and Hospital and ASHIC Foundation. The age pattern and distribution of cancer types were analysed and the incidence rates were calculated.

**Results:**

The age-standardised incidence rate was 7.8 per million person-years for children (0–14 years) in the last time period (2011–2014). Retinoblastoma (25 %) and leukaemia (18 %) were the most common childhood cancers. For adolescents (15–19 years), the age-specific incidence rate was 2.1 per million person-years in the same time period. Most common adolescent cancers were malignant bone tumours (38 %), germ cell and gonadal tumours (17 %), and epithelial tumours (16 %). There were more boys affected (M: F ratio 2.0 in children and 1.4 in adolescents) than girls.

**Conclusion:**

Cancer incidences were lower than expected most likely due to a low level of awareness about cancer among clinicians and the population, inadequate access to health care, lack of diagnostic equipment and incomplete recording of cases. Improvements on different levels should be made to get a better epidemiologic insight and to detect cancer earlier resulting in a better outcome for affected children and adolescents.

## Background

Childhood cancers are neglected in developing countries, even though approximately 84 % of the cancer cases under 15 years old occur in the low-income and middle-income countries (LMICs) [1]. Because of decreased infant mortality rates in developing countries resulting from better management of infectious diseases and current population growth, the number of childhood cancer is expected to increase by 30 % by 2020 [2].

Due to the diversity and scarcity of childhood cancer cases, conducting any epidemiological surveillance is often challenging, especially for LMICs. For these countries, where approximately 83 % of the world population is living, very limited basic epidemiological information is available [1]. The lack of basic epidemiological information on childhood malignancies hinders the understanding of the spectrum of childhood malignancies and also the efforts to set up cancer control strategies, to improve cancer care and the clinical outcomes for affected children in these countries.

In Bangladesh, the overall cancer burden including adolescent and childhood cancer is largely unknown due to the nonexistence of (population-based) cancer registries [3, 4]. The proportion of childhood cancers is expected to be high in Bangladesh because of the young population structure- about 30 % (47.4 million) of the population is under 15 years old [5]. Based on the estimated childhood cancer incidence (<15 years) of LMICs and India (102 and 124 per million person-years respectively), 5500–6700 new cases are expected each year [6, 7]. The number of pediatric cancer cases are expected to increase since Bangladesh has significantly reduced the childhood mortality rate by 71 % compared to 1990s due to better management of infectious diseases [8]. For the whole country there are only four main public hospitals (two recently introduced), which are specialized in pediatric oncology. The overall healthcare system including cancer diagnosis, treatment and management encounters severe shortage of infrastructure and trained health manpower [3]. Approximately 500 hospital beds are currently dedicated for cancer patients (both adult and children) in Bangladesh [9] and only fifteen trained pediatric hematologists/oncologists for dealing with pediatric cancers [personal communication]. This study aims to provide a comprehensive recent overview on childhood and adolescent cancers in Bangladesh, which would contribute to the understanding of epidemiologic characteristics and provide a basis for the future strategies to deal with childhood and adolescent cancers.

## Methods

Data on malignant neoplasms in patients aged less than 20 years old diagnosed between 2001 and 2014 in Bangladesh were collected by the National Institute of Cancer Research and Hospital (NICRH) and the ASHIC (A shelter for helpless ill children), a Foundation for childhood cancer. Note that, the ASHIC Foundation started registering childhood cancer cases since 2001. The pediatric oncology department of the NICRH was introduced in 2008. Before that, childhood cancer patients were treated under medical oncology department at NICRH as well as other public and private hospitals. The ASHIC Foundation is a non-governmental organization whose purpose is to support childhood cancer patients and their families in Bangladesh. They provide housing during treatment, follow-ups in Dhaka city, palliative care service and psychological counselling support [10]. This support is very important because most parents face immense difficulties when their child is diagnosed with cancer. For instance, these include travel costs, managing accommodation in Dhaka city and high treatment costs. The ASHIC Foundation also registers childhood cancer cases for specialized tertiary level hospitals outside of Dhaka city. It is important to mention here that Bangladesh is a lower middle-income country with a population of over 160 million [5], where approximately 72 % of the citizens live in the resource-limited areas, but cancer care facilities are located in the big cities, mostly in Dhaka, the capital. There is no organized referral system in Bangladesh. Generally local practitioners suggest the parents to bring their children to the specialized centre for better treatment. However, in most cases, parents decide themselves to consult with the experts of the specialized centres when local practitioners could not manage the patients properly.

Clinical observations and histological examinations were the basis of diagnosis for all collected cancer cases. Blood counts, peripheral blood films and bone marrow aspiration were used for the morphological diagnosis of leukaemia. Lymph node biopsies were used to diagnose lymphomas. Fine needle aspiration cytology or tissue biopsies were used for solid tumours. All cases were categorized according to the International Classification of Childhood Cancer (ICCC) [11]. Results were provided for all neoplasms combined as well for the main 12 ICCC diagnostic groups and the belonging subgroups for two age groups (0–14 and 15–19 years). The whole study period was divided into three time periods based on the number of collected cases: 2001–2006, 2007–2010, and 2011–2014.

### Data quality

**Fig. 1:**
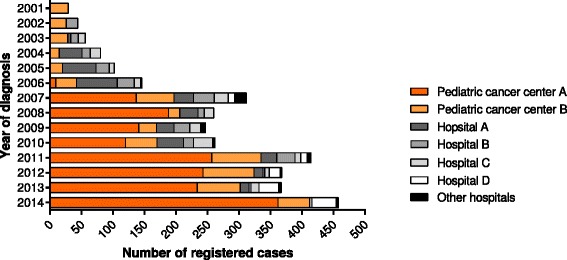
Distribution of recorded cases by hospitals, 2001–2014

Due to the lack of systematic and effective recording systems of medical records in public hospitals, duplicated and re-enrolled cases were highly expected. A patient might have visited the same hospital or different hospitals several times. Such duplicates were excluded for the analysis based on the following variables: name, gender, age at admission, year of first admission, type of cancer diagnosed and geographic location in Bangladesh (e.g., home district of the patient). Out of 3778 cases collected, 635 cases were duplicates (16.8 % of all collected cases). In the final dataset, 3143 cases were included where NIRCH and ASHIC Foundation contributed1,690 and 1453 cases respectively. Patients registered by the ASHIC Foundation were diagnosed in 20 different tertiary hospitals mostly located in Dhaka, except two hospitals outside of Dhaka city. Most cases (72 %) were derived from the two main specialized pediatric oncology centres in Dhaka (Fig. 1) and were mainly diagnosed in children aged under 15 years (93 %). Data cleaning and validation were performed by seven researchers, two pediatric oncologists, one epidemiologist and one statistician. The study protocol was ethically approved by the Ethical Review Committee (ERC) of National Institute of Cancer Research and Hospital (this is the only state-run specialized cancer hospital in Bangladesh) under the official memo no. NICRH/Ethics/2013/104. Our retrospective study was based on medical record and therefore, the issue of informed consent was waived by ERC of NICRH.

### Statistical analyses

Distribution of the 12 main ICCC diagnostic groups was given for the three study periods. In case of adolescent cancers, data was available from 2007. Because of this limitation, we considered 2001–2006 as the first time period for children and continued with two subsequent equal time periods (2007–2010 and 2011–2014) for childhood and adolescent cancers. The incidence rates were calculated as the average annual number of cases per million person-years [12]. For the population at risk the average of estimated population numbers from 2000 to 2005 was taken for the first study period 2001–2006, 2005 and 2010 for 2007–2010, and 2010 and 2015 for the last study period 2011-2014 [5]. Weights of the World standard population were used to calculate age-standardised rates (ASR) for the age group 0–14 years, and age-specific rates (Rate) were given for the age group 15–19 years. Analyses were performed using SAS software (SAS system 9.2, SAS Institute, Cary, NC).

## Results

A total of 3,143childhood and adolescent cancer cases were collected over the study period of 2001–2014 for this retrospective study. The average number of collected cases per year varied from 76 in 2001–2006 to 247 in 2007–2010 and 369 in 2011–2014. The age-standardised incidence rate was 7.8 per million children and 2.1 per million adolescents (Table 1) in 2011–2014. The sex ratio (M: F) declined from 2.5 in 2001–2006 to 2.1 in 2007–2010 and 1.9 in 2011–2014. During the first period approximately 80 % of the childhood cancer cases were leukaemias and lymphomas, while this was 51 % in 2007–2011 and about 25 % in 2011–2014. Other large changes in time were observed for retinoblastoma, malignant bone tumours, and germ cell and gonodal tumours (Table 1). In the latest period, the most common cancer types were leukaemia, retinoblastoma and malignant bone tumours. Acute lymphoblastic leukaemia (ALL) was by far the most common type of leukaemia (86 %). Half of all malignant bone tumours were osteosarcomas while 45 % was Ewing tumours. Lymphomas were the fifth most prevalent type of childhood cancer, where the proportion of non-Hodgkin lymphoma (59 %) was higher than Hodgkin lymphoma (19 %). One fourth of the epithelial tumours were nasopharyngeal carcinomas.

**Table 1:** Childhood cancer incidence (0–14 years) by period of diagnosis in Bangladesh, 2001–2014

	Period of diagnosis									
	2001–2006			2007–2010			2011–2014			
ICCC diagnostic group	N	%	ASR^a^	N	%	ASR^a^	N	%	ASR^a^	M:F ratio
Total	455		2.0	988		5.1	1474		7.8	2.0:1
I Leukaemia	269	59.1	1.2	264	26.7	1.4	271	18.4	1.4	3.2:1
ALL	226			212			234			
AML	18			38			29			
II Lymphoma	89	19.6	0.4	237	24.0	1.2	115	7.8	0.6	2.7:1
Hodgkin lymphoma	7			74			22			
Non-Hodgkin lymphoma	31			121			68			
III CNS tumours	–	–	–	5	0.5	0.03	65	4.4	0.3	1.5:1
Ependymoma	–			–			15			
Astrocytoma	–			–			16			
Medulloblastoma	–			–			16			
IV Neuroblastoma	17	3.7	0.08	24	2.4	0.1	54	3.7	0.3	1.0:1
V Retinoblastoma	6	1.3	0.03	74	7.5	0.4	374	25.4	2.1	1.8:1
VI Renal tumours	27	5.9	0.2	95	9.6	0.5	90	6.1	0.5	2.1:1
Nephroblastoma	25			87			89			
VII Hepatic tumours	8	1.8	0.04	13	1.3	0.07	29	2.0	0.2	3.8:1
Hepatoblastoma	8			13			29			
VIII Bone tumours	2	0.4	0.01	60	6.1	0.3	175	11.9	0.9	1.6:1
Osteosarcoma	1			38			91			
Chondrosarcoma	–			–			4			
Ewing tumour	1			22			79			
IX Soft tissue sarcomas	18	4.0	0.09	45	4.6	0.2	101	6.9	0.5	2.0:1
Rhabdomyosarcoma	18			34			74			
Fibrosarcoma	–			3			14			
X Germ cell and gonadal tumours	7	1.5	0.03	117	11.8	0.6	125	8.5	0.6	1.2:1
XI Other epithelial tumours	3	0.7	0.01	36	3.6	0.2	52	3.5	0.3	2.7:1
Nasopharyngeal carcinoma	1			18			13			
XII Other and unspecified tumours	9	2.0	0.04	18	1.8	0.09	23	1.6	0.1	1.6:1

^a^ASR: Age standardised rate per 1 million person-years (World Standard Population)

Most common cancer types among adolescents were lymphomas, malignant bone tumours and germ cell and gonadal tumours in 2007–2010. Largest shifts in 2011–2014 were observed for lymphomas (−82 %), CNS tumours (+900 %) and malignant bone tumours (+178 %), which led to the following top three most common cancer types: malignant bone tumours, germ cell and gonadal tumours, and epithelial tumours (Table 2). Osteosarcomas were the most prevalent malignant bone tumour (68 %). In contrast to the children, nasopharyngeal carcinomas were less common among adolescents while three patients were surprisingly diagnosed with retinoblastoma at 15–19 years age group.

**Table 2:** Adolescent cancer incidence (15–19 years) by period of diagnosis in Bangladesh, 2001–2014

	Period of diagnosis						
	2007–2010			2011–2014			
ICCC diagnostic group	N	%	Rate^a^	N	%	Rate^a^	M:F ratio
Total	93		1.5	133		2.1	1.4:1
I Leukaemia	3	3.2	0.05	6	4.5	0.09	5.0:1
ALL	2			6			
AML	1			–			
II Lymphoma	38	40.9	0.6	7	5.3	0.1	1.3:1
Hodgkin lymphoma	13			2			
Non-Hodgkin lymphoma	25			4			
III CNS tumours	1	1.1	0.02	10	7.5	0.2	2.3:1
Ependymoma	–			–			
Astrocytoma	–			3			
Medulloblastoma	–			5			
IV Neuroblastoma	–	–	–	–	–	–	–
V Retinoblastoma	1	1.1	0.02	2	1.5	0.03	–
VI Renal tumours	3	3.2	0.05	–	–	–	–
Nephroblastoma	3			–			
VII Hepatic tumours	–	–	–	1	0.8	0.02	–
Hepatoblastoma	–			1			
VIII Bone tumours	18	19.4	0.3	50	37.6	0.8	1.9:1
Osteosarcoma	13			34			
Chondrosarcoma	–			1			
Ewing tumour	5			15			
IX Soft tissue sarcomas	6	6.5	0.1	11	8.3	0.2	0.6:1
Rhabdomyosarcoma	5			6			
Fibrosarcoma	–			2			
X Germ cell and gonadal tumours	14	15.1	0.2	22	16.5	0.4	0.4:1
XI Other epithelial tumours	7	7.5	0.1	21	15.8	0.3	4.3:1
Nasopharyngeal carcinoma	4			1			
XII Other and unspecified tumours	2	2.2	0.03	3	2.3	0.05	0.5:1

^a^Rate: Age-specific rate per 1 million person-years

**Fig. 2:**
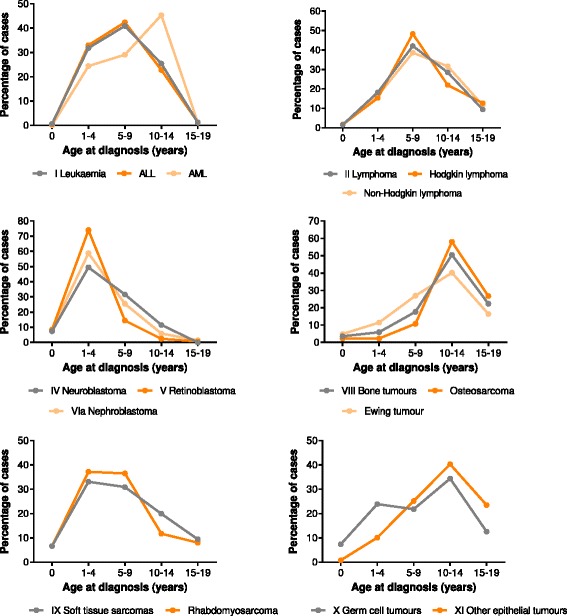
Pattern of age at diagnosis of selected childhood and adolescent cancers in Bangladesh, 2001–2014

The lowest median age at diagnosis was 3 years for retinoblastoma, renal tumours and hepatic tumours, while 12 years for malignant bone tumours. Retinoblastoma (83 %), nephroblastoma (67 %) and neuroblastoma (57 %) predominantly occurred among children aged 0–4 years (Fig. 2). Bone tumours (87 %), germ cell and gonadal tumours (44 %), and other epithelial tumours (64 %) were frequently observed among the older age groups (10–19 years). Leukaemias were mostly diagnosed in children aged 5–9 years (41 %). The same pattern was also observed forALL whileacute myeloid leukaemia (AML) was more common among children aged 10–14 years (45 %).

## Discussion

Best of our knowledge, this is the first epidemiological study that provides an overview on childhood and adolescent cancer in Bangladesh. Retinoblastoma and leukaemias were the most common childhood cancers while malignant bone tumours, germ cell and gonadal tumours, and epithelial tumours were more common among adolescents. In contrast, a single-hospital based study (*n* = 1250) showed that lymphoma was the most common childhood cancer in Bangladesh [13], while another study reported nephroblastoma/Wilmstumour being the most frequent pediatric malignancy (*n* = 70) [14]. However, the strength of this particular study is that it has collected cases from 20 different tertiary hospitals and therefore, this study provides a better representation of the overall scenario of childhood cancers in Bangladesh.

Over time, the childhood and adolescent cancer incidence has increased which is most likely due to improved awareness among clinicians, diagnostics and registration. Hence, the most recent period (2011–2014) represents the most reliable overview although the incidence rates are still low compared to India where the total childhood cancer rates varied between 38 and 124 per million person-years compared to 8 per million person-years in Bangladesh [6]. Underreporting of malignancies is well-known in resource-limited countries. Apart from inadequate access to health care, lack of professional education, infrastructure (such as advanced diagnostic facilities and imaging devices) and low level of health awareness as well as various socioeconomic factors that lead to the under-representation of cancer incidence; the presenting symptoms for some pediatric cancers (especially leukaemias) resemble with those of infections. Collecting epidemiological data wasalso encountered with huge difficulties due to lack of an adequate record keeping system in public hospitals, for which it was not possible to study e.g., treatment outcomes and survival.

Sex-specific differences in the incidence of pediatric malignancies are consistent globally. Male predominance is a common phenomenon for many childhood cancers. In developed countries, the sex ratio of boys to girls is about 1.1 ~ 1.34 [15], where some cancers including nephroblastoma and retinoblastoma generally exhibit slightly female preponderance [16]. The overall proportion of cancers was much higher in males than females in Bangladesh. For some cancers (leukaemias and hepatoblastoma), the male predominance was noted to be more than three times higher among Bangladeshi boys, while the sex ratio was almost equal for neuroblastoma, and germ cell and gonadal tumours.

This retrospective study revealed that retinoblastoma was the most frequent (25 %) childhood cancer in Bangladesh in 2011–2014. The numbers of retinoblastoma patients are generally higher in developing countries as they have high birth rates, such as in Asia and Africa [17]. Most cases (83 %) occurred in children younger than 5 years old (Fig. 2) with a median age of 3 years. Even though it is generally very uncommon after the age of 10, we have noted that about 3 % of all retinoblastoma cases were aged between 10 and 19 years. This could be due to a delayed diagnosis, which is common in developing countries [18, 19]. Retinoblastoma is a curable tumour in more than 90 % of cases, if it is detected at early stages [20]. As compared to other malignancies, the early signs of retinoblastoma are easily detectable if healthcare professionals as well as parents are aware of this malignant disease. A retinoblastoma education programme in Honduras has shown to reduce the proportion of advanced stages significantly. However, it was not successful in improving treatment compliance [21].

Leukaemias were the second most common childhood malignancy (18 %) in Bangladesh in 2011–2014. However, during the whole study period (2001–2014), leukaemias constituted most cases (28 %). The proportion of leukaemias varies across different countries ranging from 27 to 35 % [6, 15, 21, 22]. In US, for instance, leukaemias account for 31 % of all pediatric cancers, while it is approximately 37 % in Kolkata, a neighboring Indian state and nearly 26 % in Pakistan with similar culture and socioeconomic structures to Bangladesh [22, 23]. ALL comprised of the major proportion (84 %) of childhood leukaemia (Table 1) between 2001 and 2014. As mentioned earlier about under-reporting, a recent population-based study has shown that nearly 15–35 % of ALL cases go unreported [24]. Reasonably, taking all these issues together, the proportion of leukaemias would be significantly much higher in Bangladesh than our present findings. The presenting mean age of leukaemia patients in South Asian countries (Bangladesh, India and Pakistan) was found to be higher (6–7 years) than those of Western countries where incidence peak was between 0 and 4 years [16, 25–27]. Interestingly, the similar age distribution of leukaemia was also noted among South Asian population in UK [28].

In high-income countries, brain/CNS tumours are the second most pediatric cancer comprising 20–27 % of all cases, whereas lymphomas are the distant third childhood malignancy [16, 29–31]. However, it has been found that lymphomas were the fifth most frequently diagnosed cancer (7.8 %) in Bangladeshi children and CNS tumours were even more less common (4.4 %), ranked eighthamong of all childhood cancers. Very low incidence rates of CNS tumours in low-income countries including Bangladesh is likely associated with the lack of modern diagnostic facilities [7]. In cases of lymphomas, a similar pattern was also noted in India and Pakistan but the pattern of NHL and HD was opposite to this study [6, 22]. We observed that there was a higher proportion of NHL (about 70 %) in comparison to HD, a pattern similar to developed world. The age distribution was similar for NHL and HD; about 80 % of lymphoma cases were diagnosed in children aged 5–14 years with a median age between 7 and 8 years [Fig. 2]. However, HD is usually rare among children younger than 10 years, but one of the most common cancers among adolescents (15–19 years) in industrialized countries [16, 32].

Malignant bone tumours were the most common type of cancer among adolescents and ranked third among childhood cancer with a median age of 12 years. Osteosarcoma (58 %) and Ewing sarcoma (40 %) were the two most common types of malignant bone cancers. The age-specific distribution pattern of osteosarcoma and Ewing sarcoma showed that they were rare before the age of five years and the proportion increased with ages throughout childhood; both peaked at the ages of 10 to 13 years. This age pattern resembles with that of developed countries [16].

## Conclusion

This is the first study which provides an overview on the distribution of pediatric cancers in Bangladesh. Incidences are lower than expected most likely due to a low level of awareness regarding cancer among clinicians and the population, inadequate access to health care, lack of diagnostic equipment and incomplete recording of cases. Improvements on different levels(e.g., training more pediatricians about symptoms of childhood and adolescent cancer, availability of diagnostic equipment, good documentation of medical information in hospitals) should be implemented to get a better insight into the size of this ‘health problem’ and to detect cancer earlier, which will subsequently result in a better outcome for affected children and adolescents.

## Abbreviations

ALL: acute lymphoblastic leukaemia
AML: acute myeloid leukaemia
CNS: central nervous system
HD: Hodgkin’s disease
NHL: Non-Hodgkin lymphoma
NICRH: National Institute of Cancer Research and Hospital

## References

[1] Magrath I, Steliarova-Foucher E, Epelman S, Ribeiro RC, Harif M, Li CK, Kebudi R, Macfarlane SD, Howard SC. Paediatric cancer in low-income and middle-income countries. Lancet Oncol. 2013;14(3):e104–16. 10.1016/S1470-2045(13)70008-1 23434340

[2] Rodriguez-Galindo C, Friedrich P, Morrissey L, Frazier L (2013). Global challenges in pediatric oncology. Curr Opin Pediatr.

[3] Hossain MS, Ferdous S, Karim-Kos HE (2014). Breast cancer in South Asia: a Bangladeshi perspective. Cancer Epidemiol.

[4] Hossain MS, Iqbal MS, Khan MA, Rabbani MG, Khatun H, Munira S, Miah MM, Kabir AL, Islam N, Dipta TF et al. Diagnosed hematological malignancies in Bangladesh - a retrospective analysis of over 5000 cases from 10 specialized hospitals. BMC Cancer. 2014;14:438. 10.1186/1471-2407-14-438 PMC4063230 24929433

[5] World Population Prospects. 2015 Revision: http://esa.un.org/unpd/wpp/Download/Standard/Population/. Accessed 14 Feb 2016.

[6] Arora RS, Eden TO, Kapoor G (2009). Epidemiology of childhood cancer in India. Indian J Cancer.

[7] Howard SC, Metzger ML, Wilimas JA, Quintana Y, Pui CH, Robison LL, Ribeiro RC. Childhood cancer epidemiology in low-income countries. Cancer. 2008;112(3):461–72. 10.1002/cncr.23205 18072274

[8] World Bank. http://data.worldbank.org/indicator/SH.DYN.MORT. Accessed 14 Feb 2016.

[9] Hussain SA, Sullivan R (2013). Cancer control in Bangladesh. Jpn J Clin Oncol.

[10] Islam A, Eden T (2013). Brief report on pediatric oncology in Bangladesh. South Asian J Cancer.

[11] Steliarova-Foucher E, Stiller C, Lacour B, Kaatsch P (2005). International classification of childhood cancer, third edition. Cancer.

[12] Boyle D, Parkin DM. Statistical methods for registries. In: Cancer registration: principles and methods. International Agency for Research on Cancer publication no.95. 1991. https://www.iarc.fr/en/publications/pdfs-online/epi/sp95/sp95-chap11.pdf. Accessed 14 Feb 2016.

[13] Jabeen S, Haque M, Islam MJ, Talikder MH (2010). Profile of paediatric malignancies: a five year study. J Dhaka Med Coll.

[14] Hasan GZ, Hossain AKMZ, Amin MR, Siddiqui MTH, Islam KMD (2011). Pattern of childhood malignant tumour in the Paediatric Surgery Department of Bangabandhu Sheikh Mujib Medical University. BSMMU J.

[15] Bhopal SS, Mann KD, Pearce MS (2012). Registration of cancer in girls remains lower than expected in countries with low/middle incomes and low female education rates. Br J Cancer.

[16] Ward E, DeSantis C, Robbins A, Kohler B, Jemal A (2014). Childhood and adolescent cancer statistics, 2014. CA Cancer J Clin.

[17] Dimaras H, Kimani K, Dimba EA, Gronsdahl P, White A, Chan HS, Gallie BL. Retinoblastoma. Lancet. 2012;379(9824):1436–46. 10.1016/S0140-6736(11)61137-9 22414599

[18] Chantada G, Fandino A, Manzitti J, Urrutia L, Schvartzman E (1999). Late diagnosis of retinoblastoma in a developing country. Arch Dis Child.

[19] Rodrigues KE, Latorre Mdo R, de Camargo B (2004). Delayed diagnosis in retinoblastoma. J Pediatr (Rio J).

[20] Abramson DH, Beaverson K, Sangani P, Vora RA, Lee TC, Hochberg HM, Kirszrot J, Ranjithan M. Screening for retinoblastoma: presenting signs as prognosticators of patient and ocular survival. Pediatrics. 2003;112(6 Pt 1):1248–55. 10.1542/peds.112.6.1248 14654593

[21] Leander C, Fu LC, Pena A, Howard SC, Rodriguez-Galindo C, Wilimas JA, Ribeiro RC, Haik B. Impact of an education program on late diagnosis of retinoblastoma in Honduras. Pediatr Blood Cancer. 2007;49(6):817–9. 10.1002/pbc.21052 17009236

[22] Badar F, Mahmood S, Zaidi A, Bhurgri Y (2009). Age-standardized incidence rates for childhood cancers at a cancer hospital in a developing country. Asian Pac J Cancer Prev.

[23] Datta K, Choudhuri M, Guha S, Biswas J (2011). Childhood cancer burden in part of eastern India--Population Based Cancer Registry data for Kolkata (1997–2004). Asian Pac J Cancer Prev.

[24] Azevedo-Silva F, Reis Rde S, Santos Mde O, Luiz RR, Pombo-de-Oliveira MS (2009). Evaluation of childhood acute leukemia incidence and underreporting in Brazil by capture-recapture methodology. Cancer Epidemiol.

[25] Bajel A, George B, Mathews V, Viswabandya A, Kavitha ML, Srivastava A, Chandy M. Treatment of children with acute lymphoblastic leukemia in India using a BFM protocol. Pediatr Blood Cancer. 2008;51(5):621–5. 10.1002/pbc.21671 18688848

[26] Fadoo Z, Nisar I, Yousuf F, Lakhani LS, Ashraf S, Imam U, Zaheer J, Naqvi A, Belgaumi A. Clinical features and induction outcome of childhood acute lymphoblastic leukemia in a lower/middle income population: a multi-institutional report from Pakistan. Pediatr Blood Cancer. 2015;62(10):1700–8. 10.1002/pbc.25583 25982135

[27] Sazawal S, Gurbuxani S, Bhatia K, Khattar A, Raina V, Arya LS, Vats T, Magrath I, Bhargava M. Incidence, clinical characteristics and early treatment outcome in Indian patients of childhood acute lymphoblastic leukemia with ALL-1 gene rearrangement. Leuk Res. 2001;25(8):693–8. 10.1016/s0145-2126(01)00007-8 11397475

[28] McKinney PA, Feltbower RG, Parslow RC, Lewis IJ, Glaser AW, Kinsey SE (2003). Patterns of childhood cancer by ethnic group in Bradford, UK 1974–1997. Eur J Cancer.

[29] Baade PD, Youlden DR, Valery PC, Hassall T, Ward L, Green AC, Aitken JF. Trends in incidence of childhood cancer in Australia, 1983–2006. Br J Cancer. 2007;102(3):620–6. 10.1038/sj.bjc.6605503 PMC2822940 20051948

[30] Hung GY, Horng JL, Lee YS, Yen HJ, Chen CC, Lee CY (2014). Cancer incidence patterns among children and adolescents in Taiwan from 1995 to 2009: a population-based study. Cancer.

[31] Kaatsch P, Steliarova-Foucher E, Crocetti E, Magnani C, Spix C, Zambon P (2006). Time trends of cancer incidence in European children (1978–1997): report from the Automated Childhood Cancer Information System project. Eur J Cancer.

[32] Clavel J, Steliarova-Foucher E, Berger C, Danon S, Valerianova Z (2006). Hodgkin’s disease incidence and survival in European children and adolescents (1978–1997): report from the Automated Cancer Information System project. Eur J Cancer.

